# Transcriptomic and genomic profiling of early-stage ovarian carcinomas associated with histotype and overall survival

**DOI:** 10.18632/oncotarget.26225

**Published:** 2018-10-12

**Authors:** Hanna Engqvist, Toshima Z. Parris, Elisabeth Werner Rönnerman, Elin M.V. Söderberg, Jana Biermann, Claudia Mateoiu, Karin Sundfeldt, Anikó Kovács, Per Karlsson, Khalil Helou

**Affiliations:** ^1^ Department of Oncology, Institute of Clinical Sciences, Sahlgrenska Cancer Center, Sahlgrenska Academy at University of Gothenburg, Gothenburg, Sweden; ^2^ Sahlgrenska University Hospital, Department of Clinical Pathology and Genetics, Gothenburg, Sweden; ^3^ Department of Surgery, Institute of Clinical Sciences, Sahlgrenska Cancer Center, Sahlgrenska Academy at University of Gothenburg, Gothenburg, Sweden; ^4^ Department of Obstetrics and Gynecology, Institute of Clinical Sciences, Sahlgrenska Academy at University of Gothenburg, Gothenburg, Sweden

**Keywords:** ovarian cancer, diagnostic biomarker, prognostic biomarker, genomics

## Abstract

Ovarian cancer is the most lethal gynecological malignancy in the western world. Despite recent efforts to characterize ovarian cancer using molecular profiling, few targeted treatment options are currently available. Here, we examined genetic variants, fusion transcripts, SNP genotyping, and gene expression patterns for early-stage (I and II) ovarian carcinomas (n=96) in relation to clinicopathological characteristics and clinical outcome, thereby identifying novel genetic features of ovarian carcinomas. Furthermore, mutation frequencies of specific genetic variants and/or their gene expression patterns were associated with histotype and overall survival, *e.g. SLC28A2* (mucinous ovarian carcinoma histotype), *ARCN1* (low expression in 0-2 year survival group), and tumor suppressor *MTUS1* (mutation status and overall survival). The long non-coding RNA *MALAT1* was identified as a highly promiscuous fusion transcript in ovarian carcinoma. Moreover, gene expression deregulation for 23 genes was associated with tumor aggressiveness. Taken together, the novel biomarkers identified here may improve ovarian carcinoma subclassification and patient stratification according to histotype and overall survival.

## INTRODUCTION

Recent advances in our understanding of ovarian carcinoma contributed to the reclassification of the disease into five major histotypes (high-grade serous (HGSC), low-grade serous (LGSC), endometrioid (EC), mucinous (MC) and clear cell (CCC) carcinomas) based on differences in origin, morphology, and clinical and biological behavior [1–3]. Standard treatment options for ovarian carcinoma are currently limited to surgical cytoreduction, followed by platinum-based chemotherapy, despite the recent introduction of new promising treatment options, *e.g.* poly (ADP-ribose) polymerase (PARP) inhibitors as a targeted therapy for carriers of genetic variants in *BRCA1/2*, the use of antiangiogenic agents in combination with first-line treatment or as maintenance treatment, and the administration of intraperitoneal chemotherapy [4]. The majority of ovarian cancer patients are still currently treated with conventional treatment based on tumor stage and grade, regardless of histotype or other biological characteristics. Unfortunately, there are no alternative treatment regimens alone that have been proven to be superior to conventional therapy, which in itself is inadequate since *e.g.* the overall 5-year survival rate is below 50% and the majority of advanced-stage ovarian cancer patients develop recurrences [4–7]. Consequently, there is a profound need for novel biomarkers with improved prognostic and diagnostic value that may guide the selection of therapeutic targets and thereby improve personalized medicine based on an individual ovarian carcinoma patient's clinicopathological and tumor characteristics [8].

Since the introduction of the revised 2014 World Health Organization (WHO) criteria for histotype diagnoses, few efforts have been made to identify novel molecular biomarkers that stratify ovarian carcinomas according to clinical outcome and the current histotypes. Surprisingly, relatively few studies have characterized histotype-specific genetic features in early-stage ovarian carcinomas (I and II) [9–11]. In addition, few studies have previously been performed on early-stage ovarian carcinomas to identify novel prognostic biomarkers [12]. One reason may be that the majority of all ovarian carcinomas are diagnosed at advanced stages III and IV [13].

Cancer is a complex disease comprised of genetic alterations that are accumulated during cancer development and progression. Although early-stage high grade ovarian carcinoma is rare, a recent report showed that early- and late-stage HGSC share many acquired genetic aberrations [14]. Therefore, early-stage tumors were chosen for the present study since they generally present less complex genetic profiles in comparison with late-stage tumors, thereby simplifying the analysis. Here, 96 primary ovarian carcinomas corresponding to the International Federation of Gynecology and Obstetrics (FIGO) stages I and II were profiled using RNA sequencing (RNA-seq) and genome-wide SNP genotyping. The present study thereby presents a good opportunity to characterize specific events associated with ovarian carcinogenesis and disease-specific genetic aberrations. The transcriptomic and genomic data were integrated with clinicopathological features and patient clinical outcome to characterize a) genetic aberrations associated with histotype or patient survival and b) novel prognostic and diagnostic biomarkers that may improve ovarian tumor classification and patient stratification. The current study thereby identifies novel genetic profiles and constitutes an important addition to existing research.

## RESULTS

### Landscape of genetic variation reveals few potentially deleterious variants in early-stage ovarian carcinoma

**Figure 1:**
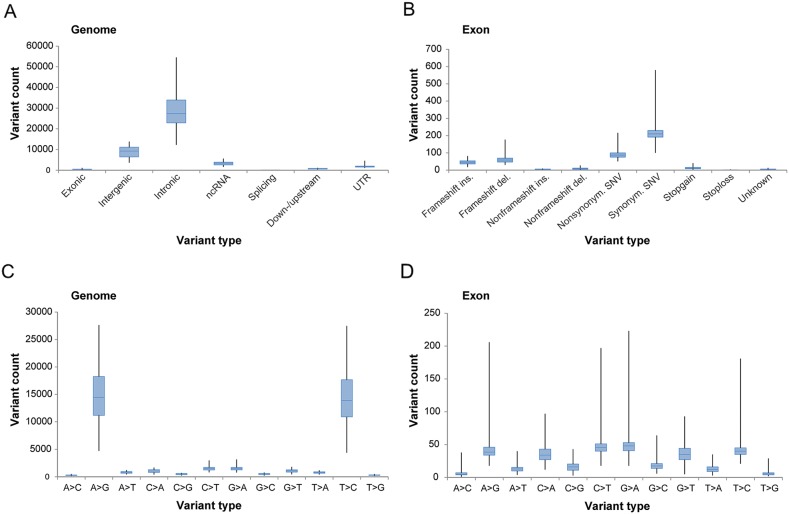
Overview of tumor-specific genetic variation derived from whole-transcriptome RNA-seq Box plots illustrating the number of genetic variants **(A-B)** in genomic and exonic regions based on location within the genome and variant type within the exonic region, and the number of base-pair substitutions **(C-D)** in genomic and exonic regions, present in the study cohort.

After removing common genetic variants found in the normal human population, the mean number of genomic and coding (exonic) variants per tumor was 44,138.3±1,240.3 (±SEM; range, 19,884-80,024) and 442.3±11.9 (range, 203-1,146), respectively. The variants are hereinafter termed tumor-specific variants. The number of tumor-specific variants was most prevalent within intronic regions of the genome, followed by variants within intergenic and non-coding RNA (ncRNA) regions, respectively (Figure 1A). In exonic regions, tumor-specific coding variants spanned 11,529 different genes and the most frequent variant type was synonymous single-nucleotide variants (SNVs) followed by nonsynonymous SNVs (Figure 1B). Base-pair substitutions associated with A>G (39.4%) and T>C (38.4%) were most prevalent in the genomic regions and G>A (15.3%), C>T (15.2%), T>C (12.8%) and A>G (12.6%) were most prevalent in the exonic regions (Figure 1C–1D).

After conservative filtering, 8,187 tumor-specific coding variants (frameshift insertion, frameshift deletion, stopgain, or stoploss) were identified, which were predicted to have a disruptive effect on protein function (classified as potential deleterious variants, *e.g.* resulting in protein truncation, gain/loss of function or nonsense mediated decay). Among the 8,187 variants, potential deleterious variants were identified in 26 genes in at least 30% of the samples. Frameshift insertion and deletion were more prevalent than stopgain and stoploss and no significant differences in variant types were detected between the different histotypes and survival groups.

**Figure 2:**
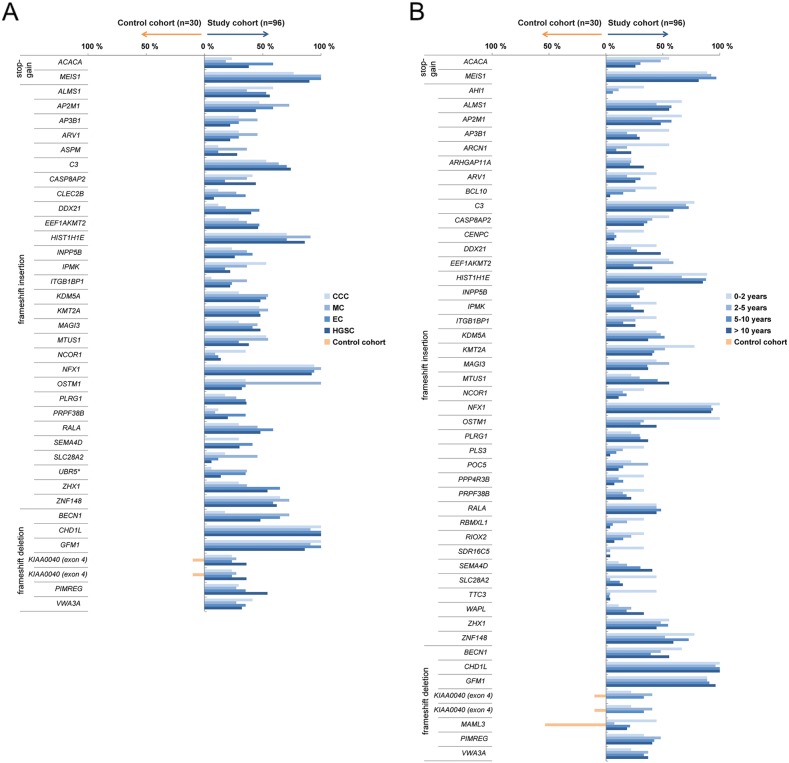
Recurrent deleterious variants Blue bars depict recurrent deleterious variants present in at least 30% of at least one of the histotype groups **(A)** or survival groups **(B)** across the study cohort. Orange bars designate deleterious variants found in the control cohort. Genes known to be associated with cancer, according to the COSMIC database, are indicated by ^*^.

The mutation frequency of recurrent deleterious variants, *i.e.* deleterious variants present in at least 30% of the patients, were also assessed for each histotype (HGSC, EC, MC, CCC) and survival group (0-2 years, 2-5 years, 5-10 years or >10 years; Figure 2). Thirty-eight and 49 recurrent deleterious variants were identified in the histotype and survival groups, respectively. To identify tumor-specific variants in ovarian carcinoma, the presence of the identified recurrent deleterious variants were evaluated in normal ovarian tissue samples (control cohort). All of the recurrent deleterious variants significantly differed in mutation rates between the study cohort and the normal controls (Supplementary Table 1A-1B). Four recurrent deleterious variants corresponding to the *CHD1L* (99.0%), *GFM1* (91.7%), *MEIS1* (90.6%) and *NFX1* (93.8%) genes were found in the majority of the tumor samples in the study cohort, but in none of the normal controls. Frameshift insertion in the *UBR5* gene was the only deleterious variant to be found in the COSMIC database. Only three deleterious variants in the *KIAA0040* (study cohort 31.3%, control cohort 10%) and *MAML3* (study cohort 18.8%, control cohort 53.3%) genes were also present in the control cohort.

Pathway analysis with Ingenuity Pathway Analysis (IPA), performed using genes corresponding to the potential deleterious variations, revealed an association with a number of cancer-related pathways. Biological processes significantly associated with the histotype and survival groups included cell cycle, cell death and survival, cell morphology, cellular development, cellular movement and gene expression.

### Histotype and survival group specific mutation frequency and its impact on gene expression patterns

**Figure 3:**
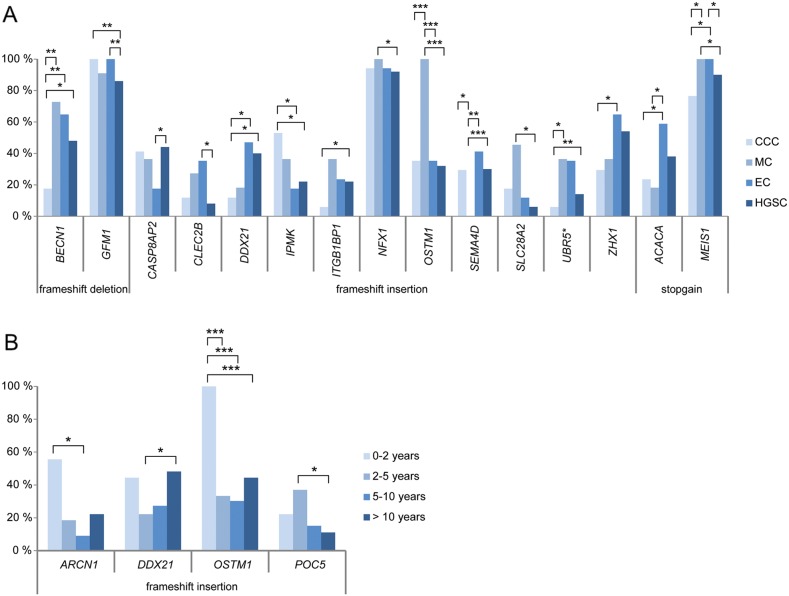
Significant mutation rate differences between histotype **(A)** and survival groups **(B)**. Bar plots showing the genes comprising recurrent deleterious variants which show at least one significant difference (^*^P<0.05, ^**^P<0.01, ^***^P<0.001) in mutation rates between any of the histotype or survival groups.

The mutation frequency for several deleterious genetic variants also varied between the different histotypes and survival groups (Figure 2). Deleterious variants in the *CASP8AP2* (HGSC 44.0%, EC 17.6%) and *CLEC2B* (HGSC 8.0%, EC 35.3%) genes showed significantly higher and lower mutation rates in HGSC compared to EC, respectively. In addition, the *OSTM1* (HGSC 32.0%, EC 35.3%, MC 100%, CCC 35.3%) and *SEMA4D* (HGSC 30.0%, EC 41.2%, MC 0%, CCC 29.4%) genes showed significantly higher and lower mutation rates in MC compared to the other histotype groups, respectively. Genetic variants in the *ACACA* (EC 58.8%, MC 18.2%, CCC 23.5%) gene were more prevalent in the EC compared to both the CCC and MC groups. Deleterious variants in the *IPMK* (HGSC 22.0%, EC 17.6%, CCC 52.9%) gene were more prevalent in the CCC compared to the HGSC and EC groups, lower mutation rates in the *UBR5* (HGSC 14.0%, MC 36.4%, CCC 5.9%) gene for CCC compared to the HGSC and MC groups, and lower in the *BECN1* (HGSC 48.0%, EC 64.7%, MC 72.7%, CCC 17.6%) gene for CCC compared to the other histotype groups, respectively. Lastly, the mutation rate for the *SLC28A2* gene was significantly higher in MC compared to HGSC (HGSC 6.0%, MC 45.5%). With regard to the four survival groups, deleterious variants in the *OSTM1* gene (0-2 years 100%, 2-5 years 33.3%, 5-10 years 30.3%, >10 years 44.4%) showed significantly higher mutation rates in the 0-2 year survival group compared to the other survival groups (Figure 3, Supplementary Table 1A-1B).

Subsequently, differences in gene expression patterns for the potential deleterious variants were evaluated between the histotype and survival groups. The majority of the genes were differentially expressed in the different histotype (n=33, 89.2%) and survival groups (n=43, 89.6%) in comparison with the control cohort (Supplementary Table 2A-B). In addition, the expression of several genes also correlated with specific histotypes or survival groups. The *AP2M1*, *GFM1*, *HIST1H1E* and *ZNF148* genes were significantly overexpressed and the *BECN1* and *NCOR1* genes significantly underexpressed in HGSC compared to the other histotypes. The *SEMA4D* gene was significantly overexpressed in the EC histotype compared to MC samples and *ARV1* was significantly underexpressed in CCC in comparison with the other histotypes. Furthermore, *ALMS1* was significantly underexpressed in the 0-2 year survival group compared to the 2-5 year and >10 year survival groups, while *VWA3A* was significantly underexpressed in the 0-2 year survival group compared with the 2-5 year and >10 year survival groups (Supplementary Table 2A-B).

**Figure 4:**
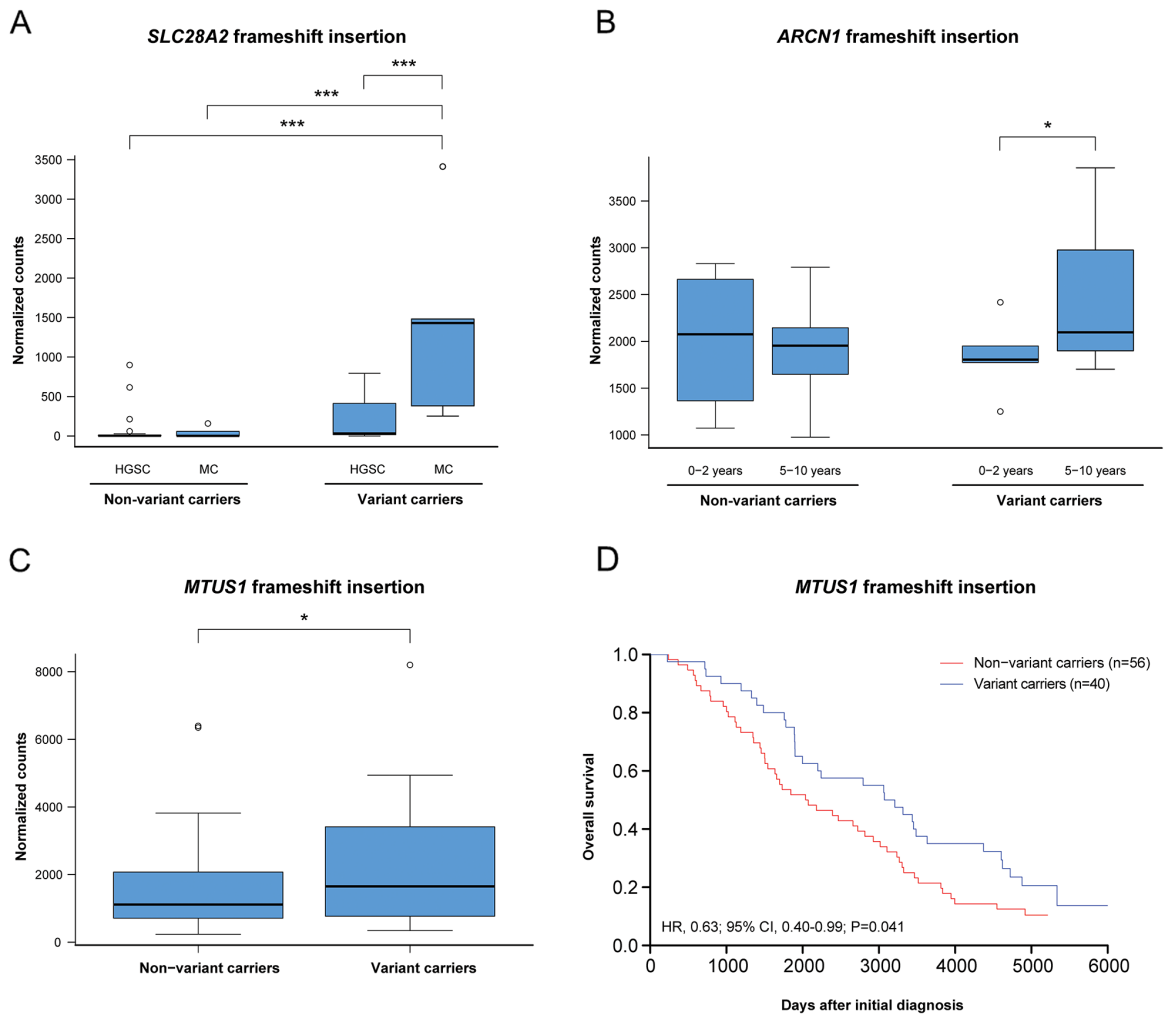
The effect of mutation status on gene expression and overall survival **(A)** Frameshift insertion in the gene *SLC28A2* caused a significant mutation and histotype-specific effect on gene expression. **(B)** Frameshift insertion in the gene *ARCN1* showed a significant differential gene expression patterns between the 0-2 years and 5-10 years survival groups. **(C-D)** In the gene *MTUS1*, a significant difference in gene expression and overall survival was seen for variant carriers comprising the frameshift insertion. Significance is indicated by ^*^ *P*<0.05, ^**^P<0.01, ^***^P<0.001.

To determine whether mutation status had an effect on the gene expression patterns of the potential deleterious variants, the expression levels for tumors harboring (variant carriers) or lacking (non-variant carriers) the recurrent deleterious variant were compared. In total, 15 of the 52 (28.8%) different genes containing deleterious variants (*AP2M1*, *ARHGAP11A*, *C3*, *CASP8AP2*, *HIST1H1E*, *IPMK*, *ITGB1BP1*, *MAGI3*, *MAML3*, *MTUS1*, *SDR16C5*, *SLC28A2*, *TTC3*, *VWA3A*, *ZHX1*) identified in either the histotype or survival groups showed a significant difference in gene expression patterns between variant and non-variant carriers. Differential gene expression was also evaluated for deleterious variants showing significant differences in mutation rates within the histotype (15/38 variants) or survival groups (4/49 variants; Figure 3). In at least one of the histotype comparisons, *BECN1*, *CASP8AP2*, *GFM1*, *IPMK*, *MEIS1*, *SEMA4D*, *SLC28A2* and *UBR5* were differentially regulated between variant and non-variant carriers. Differences in gene expression patterns were predominantly dependent on histotype and not mutation status. However, the *SLC28A2* gene showed a significant difference in gene expression patterns between variant and non-variant carriers in the MC histotype. In addition, the *ARCN1*, *OSTM1* and *POC5* genes were differentially regulated between variant and non-variant carriers in at least one of the survival group comparisons, and the *ARCN1* gene was differentially regulated between variant carriers in the 0-2 year and 5-10 year survival groups. Moreover, frameshift insertion in *MTUS1* was the only gene among the deleterious variants showing significant differences in gene expression patterns dependent on mutation status, which also had a protective effect on overall survival (OS) compared with non-variant carriers of the gene (Figure 4). No correlation was found between disease-specific survival (DSS) and the deleterious variants.

### Analysis of fusion transcripts identified a commonly occurring fusion transcript partner

**Figure 5:**
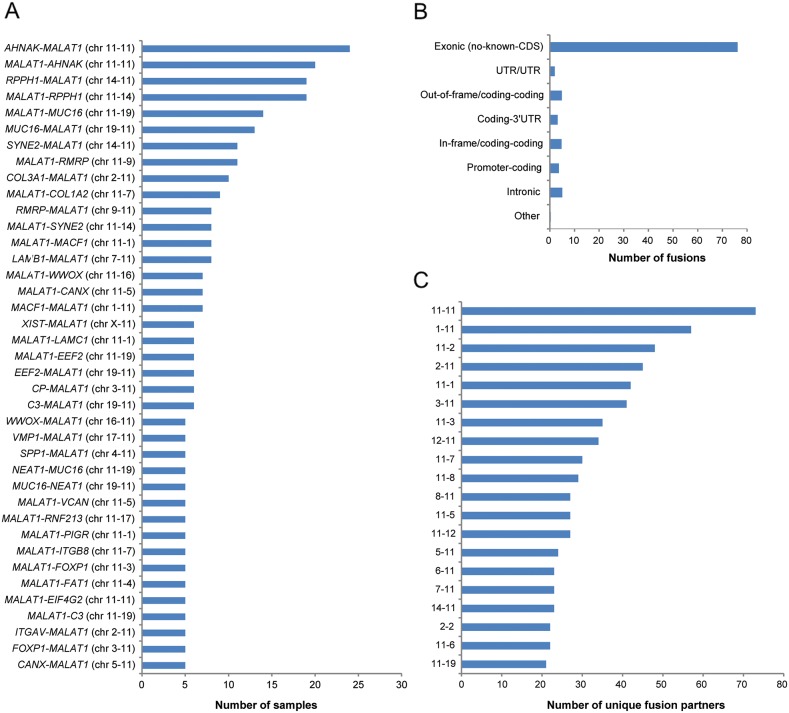
Frequency of the identified fusion transcripts across the study cohort Bar plots illustrating **(A)** the identified fusion transcripts recurrent in at least five samples in the study cohort, **(B)** the distribution of the predicted effect of the fusion transcripts across the genome, **(C)** the number of unique fusion partners in the top 20 chromosome pairs.

In total, 3,735 fusion transcripts were identified in the initial analysis, of which approximately 10.4% (n=388) of the identified fusion transcripts were flagged by FusionCatcher as having a high or very high probability of being false positives and therefore removed from further analysis. Among the remaining 3,344 fusion transcripts (mean fusion transcripts per tumor 34.7±4.4 (±SEM); range, 0-266), 1,503 unique fusion transcripts were identified. The top ten most common fusion transcripts in the study cohort were *AHNAK*-*MALAT1* (n=24), *MALAT1*-*AHNAK* (n=20), *RPPH1*-*MALAT1* (n=19), *MALAT1*-*RPPH1* (n=19), *MALAT1*-*MUC16* (n=14), *MUC16*-*MALAT1* (n=13), *SYNE2*-*MALAT1* (n=11), *MALAT1*-*RMRP* (n=11), *COL3A1*-*MALAT1* (n=10), *MALAT1*-*COL1A2* (n=9). The long non-coding RNA (lncRNA) *MALAT1* was involved in the majority of recurrent fusion transcripts found in at least five of the study cohort samples (Figure 5A). Overall, the majority (76.2%) of the fusion transcripts had one gene partner spanning non-coding exonic regions (no known coding DNA sequence (CDS)), such as *MALAT1*. A total of 675 fusion transcripts had *MALAT1* as its 5’ fusion partner and 701 fusion transcripts had *MALAT1* as its 3’ fusion partner. A small proportion (12.7%) of the fusion transcripts were predicted to be in exonic regions (out-of-frame/coding-coding (4.9%), coding-3’UTR (3.1%), in-frame/coding-coding (4.8%), promotor-coding (3.7%)), 2.0% in untranslated regions and 5.1% in intronic regions (Figure 5B). Interchromosomal fusions were more common than intrachromosomal fusions and the most abundant fusion transcripts in the study cohort were fusion events involving chromosome 11. More specifically, the most abundant intrachromosomal fusions were found on chromosome 11 and the most abundant interchromosomal fusions were found between chromosomes 1 and 11 (Figure 5C). In addition, 17 fusion gene partners (*CDKL1, EEF2K, ERBB2, HK1, IRAK2, MAST4, MASTL, OXSR1, PAK1, PI4KA, PIP4K2B, PIP5K1B, PKM, PRKDC, TJP2, TTN* and *WDFY2*) were identified as being in-frame kinases. Forty-seven percent of the fusion transcripts contained gene partners with inverted orientation and 18 reciprocal fusion transcripts (gene A-gene B, gene B-gene A) were found among the fusion transcripts present in at least four samples (n=74). The reciprocal fusion transcripts (*AHNAK-MALAT1*, *MALAT1-AHNAK*) were found in 35.4% (n=34) of the samples. *MUC16-NEAT1* (n=5) was the most commonly found fusion transcript not involving *MALAT1*. *MALAT1* and *AHNAK* were both significantly overexpressed in the study cohort compared with the control cohort. The 11q13.1 locus spanning *MALAT1* was the most commonly involved locus spanning at least one fusion gene partner (linked to 19.6% of all unique fusion transcripts).

**Figure 6:**
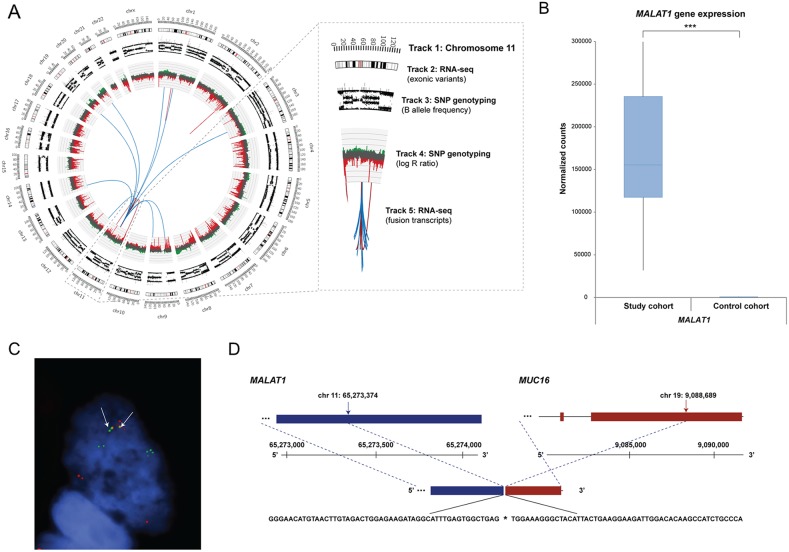
Genomic rearrangements for ovarian sample OV315, *MALAT1* gene expression pattern, and FISH validated fusion transcript **(A)** Circos plot illustrating the genomic rearrangements in the ovarian carcinoma sample OV315, containing 49 fusion transcripts. *MALAT1*, located on chromosome 11, was frequently involved in fusion events. Track 1: Chromosome cytobands, wherein the centromere is shown as a red bar. Track 2: Genetic variants in exonic regions identified with RNA-seq data are shown as dark gray bars. Track 3: B allele frequency of SNP genotyping data. Track 4: Log R ratio of SNP genotyping data, where copy number gains and losses are depicted in green and red, respectively. Track 5: Fusion transcripts identified with RNA-seq data, wherein intrachromosomal and interchromosomal gene fusions are shown in red and blue lines, respectively. **(B)** Gene expression patterns for *MALAT1* within the study cohort and control cohort (the maximum counts value for *MALAT1* within the study cohort ranged to 721344 counts). Significance is indicated by ^*^P<0.05, ^**^P<0.01, ^***^P<0.001. **(C)** FISH with interphase cells using tumor imprints for sample OV315 showing the *MALAT1* gene (green) and the *MUC16* gene (red). Yellow hybridization signals (white arrows) indicate overlapping signals representing the presence of a fusion transcript. Signals from the gene native sites are also seen as separate green and red signals. **(D)** Genomic sequence at the fusion transcript break point of *MALAT1*-*MUC16*. Chromosomal positions of the fusion break points are indicated by black arrows.

Circos plots were used to summarize the genomic rearrangements (genetic variations, copy number alterations and fusion events) in the ovarian carcinoma samples (Figure 6). SNP genotyping showed that the fusion events identified on the RNA level were frequently associated with DNA breakpoints (DNA gains or losses). Generally, DNA loss was observed more often than DNA gain. Dual-color FISH was performed on tumor touchprint preparations from at least two different tumor samples to validate fusion transcripts with *MALAT1* as the 5’ fusion partner in combination with the *AHNAK*, *RPPH1*, *MUC16*, *RMRP*, *SYNE2*, *COL3A1*, *COL1A2*, *EIFA2*, *MACF1*, *C3*, *CANX* and *XIST* as the 3’ fusion partners. FISH analysis showed intratumoral heterogeneity with few tumor cells containing the fusion transcript of interest. A validated fusion transcript between *MALAT1* and *MUC16* as well as the genomic sequence at its fusion transcript break point is shown in Figure 6.

**Figure 7:**
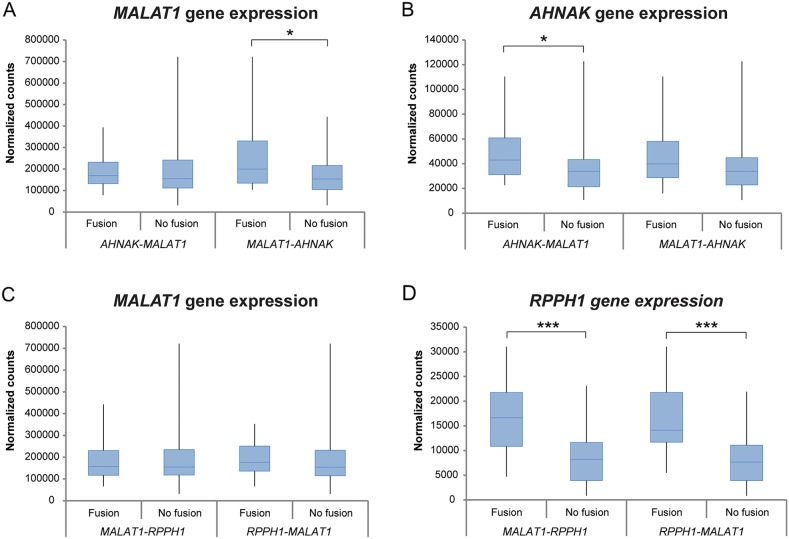
Differential gene expression analysis between the two most commonly found fusion transcripts in the study cohort Box plots showing the gene expression patterns of patients harboring the fusion transcript versus patients lacking the fusion transcript for *AHNAK-MALAT1*, *MALAT1-AHNAK*, *MALAT1-RPPH1* and *RPPH1-MALAT1* within the study group. Significant correlations are marked with ^*^P<0.05, ^**^P<0.01, ^***^P<0.001.

Differential gene expression analysis was performed between the four most commonly occurring fusion transcripts (*AHNAK*-*MALAT1*, *MALAT1*-*AHNAK*, *MALAT1*-*RPPH1* and *RPPH1*-*MALAT1*) within the study cohort (Figure 7). Significantly higher gene expression levels were seen in samples harboring specific fusion transcripts, *e.g. MALAT1* gene expression for the *MALAT1*-*AHNAK* fusion transcript, *AHNAK* gene expression for *AHNAK*-*MALAT1*, and *RPPH1* gene expression for *MALAT1*-*RPPH1* and *RPPH1*-*MALAT1*.

Oncofuse was used to identify fusion transcripts with oncogenic potential. In total, 105 of the 3,344 (3.1%) true fusion transcripts were identified across the study group as being significant driver fusion transcripts (Supplementary Table 3). Six of the most frequent fusion partners containing the *MUC16*, *AHNAK*, *SYNE2*, *COL3A1*, *COL1A2* and *MACF1* genes were found to be significant driver fusion transcripts. Five of the 17 identified kinase fusion gene partners (*EEF2K, ERBB2, MAST4, PAK1, TTN*) were also proposed by Oncofuse to be driver fusions. *MALAT1* was not identified in any significant driver fusion transcript. Pathway analysis using the significant driver fusion transcripts revealed an association with cancer-related pathways, including cell death and survival, cell morphology, cellular development, cellular growth and proliferation, and cellular movement.

### Histotype and survival group specific fusion events

The occurrence of *AHNAK-MALAT1* fusions varied among the different histotypes (HGSC (26%), LGSC (0%), EC (17.6%), MC (36.4%), CCC (23.5%)) and survival groups (0-2 years (44.4%), 2-5 years (22.2%), 5-10 years (24.2%) and >10 years (22.2%)). Moreover, the occurrence of the fusion transcripts *MALAT1-MUC16* (71.4%), *MUC16-MALAT1* (61.5%) and *COL3A1-MALAT1* (60.0%) were most commonly found in HGSC, whereas *SYNE2-MALAT1* (72.7%) in CCC.

The driver fusion transcripts were distributed across the histotype (HGSC (34.0%), EC (35.3%), MC (18.2%) and CCC (47.1%)) and survival groups (0-2 year (44.4%), 2-5 year (25.9%), 5-10 year (39.4%) and >10 year (37%)). *TTN*-*SLC7A2* was present in two ovarian carcinoma samples, OV170 and OV177. Consequently, *TTN* was also significantly underexpressed in the 2-5 year survival group compared with long-term survivors (>10 year survival group). HGSC sample OV368 harbored eight different driver fusion transcripts comprising *SPARC* as the common fusion partner, one of which was the *SPARC*-*POSTN* fusion transcript. One sample (HGSC sample OV341) had three different driver fusion transcripts involving *ERBB2*/*HER2*.

### Gene expression analysis uncover differentially expressed genes associated with tumor aggressiveness

**Figure 8:**
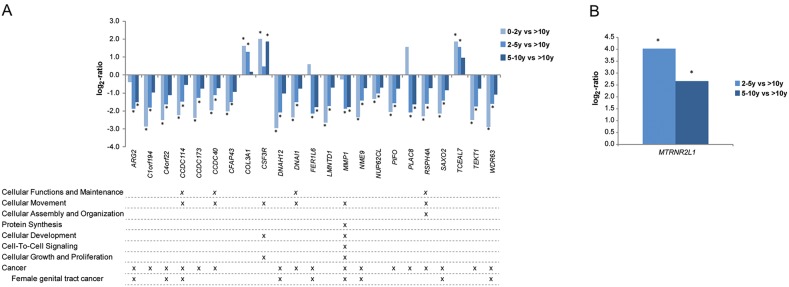
Gene expression patterns associated with tumor aggressiveness **(A)** Column bars depict overall gene expression patterns associated tumor aggressiveness when comparing short-term survivors (0-2y, 2-5y, 5-10y) with long-term survivors (>10y). Pathway analysis using IPA showed that the majority of the genes play a crucial role in cancer-related biological processes and/or associated with cancer (*P*<0.05). **(B)** Column bars showing overall gene expression patterns associated tumor aggressiveness in HGSC when comparing survivors in the survival groups 2-5 years and 5-10 years with long-term survivors (>10y), respectively. Significant log2-values (*P*<0.05) are indicated by ^*^.

Gene expression profiles for patient samples belonging to the 0-2 year, 2-5 year and 5-10 year survival groups (short-time survivors) were compared with the >10 year survival group (long-time survivors) for overall survival. In total, 127 (0-2 years vs >10 years), 134 (2-5 years vs >10 years) and 19 genes (5-10 years vs >10 years) were differentially expressed (adjusted *P*<0.05; 1.5-fold change cutoff). Twenty-three genes were recurrently deregulated in at least two of the survival group comparisons (Figure 8A). Underexpression was generally more pronounced (lower log_2_-ratio) in the 0-2 year survival group than the other short-term survival groups (adjusted *P*<0.05, 1.5-fold change cutoff). Overexpression was also more pronounced (higher log_2_-ratio) in the 0-2 year survival group. Pathway analysis showed that the differentially expressed genes were involved in cancer-related pathways such as cellular functions and maintenance, cellular movement, cellular assembly and organization, protein synthesis, cellular development, cell-to-cell signaling, and cellular growth and proliferation. In addition, *MMP1* was the only gene associated with tumor aggressiveness to play a role in at least five of the cancer-related biological processes. Three of the differentially expressed genes (*CCDC114*, *CCDC173*, *CCDC40*) are members of the *CCDC* gene family relating to cellular movement, functions and maintenance. The majority of the differentially expressed genes are known to be cancer-related, several of which are involved in female genital tract cancer.

Differential gene expression analysis was then performed by further stratifying the study cohort by both histotype and survival group. To avoid compromising the validity of the results due to small sample sizes, this analysis was only performed using three of the survival groups (2-5 years, 5-10 years and >10 years) for HGSC. The *MTRNR2L1* gene was significantly overexpressed in the 2-5 year group compared with long-term survivors (log_2_ fold change = -4.0) and 5-10 year group compared with long-term survivors (log_2_ fold change = -2.7; Figure 8B).

## DISCUSSION

Due to intertumoral heterogeneity, tumors with similar histopathology and presumed tissue of origin may have distinctly different clinical behaviors and respond differently to the same treatment. It is therefore important to improve personalized medicine using robust physiological biomarkers. Multi-omics approaches enable a comprehensive identification and evaluation of novel cancer biomarkers [15]. Here, whole-transcriptome RNA sequencing and whole-genome SNP genotyping analysis were performed on early-stage ovarian tumors in relation to clinicopathological features and clinical outcome to identify novel prognostic and/or diagnostic biomarkers, and improve histotype classification and patient stratification. This study presents an important addition to existing research due to its complete genome characterization of a large sample size of early-stage ovarian carcinoma specimens since few studies have previously characterized histotype-specific genetic features in early-stage ovarian carcinomas [9–11]. Moreover, few studies relate to complete genome studies on non-serous ovarian carcinoma histotypes [11, 16, 17] and there are few studies on early-stage HGSC. It has however been shown that early-stage HGSC show similar genetic aberrations to late-stage HGSC [14]. In the present investigation, TP53 mutation analysis showed that mutation frequencies were highest in HGSC, which is in line with frequency rates observed in late-stage HGSC [18]. Overall, we identified (a) recurrent deleterious tumor-specific genetic variants, not previously associated with cancer or tumor aggressiveness, that may have potential diagnostic value due to the absence of the variants in normal tissue, (b) expressed fusion transcripts predominantly in ncRNAs, in particular *MALAT1*, and (c) potential prognostic genes associated with tumor aggressiveness that may play a pivotal role in cancer-related processes. In summary, these results present a comprehensive characterization of ovarian carcinoma with respect to histotype and overall survival highlighting the genetic complexity of ovarian carcinomas.

Several genetic variants had high mutation rates across the entire study group in comparison with normal ovarian controls, *e.g.* recurrent deleterious variants in the *CHD1L*, *GFM1*, *MEIS1* and *NFX1* genes, suggesting specificity to cancer. Similar mutation rates were identified for these genes in a breast cancer cohort [19]. Interestingly, no genetic variants were specific for either ovarian or breast cancer suggesting similarities between the genetic variants found in both cancer forms. Furthermore, genetic variants in *CHD1L*, *GFM1*, *MEIS1* and *NFX1* have previously been reported in other cancer types (the type of genetic variant was not specified) but at lower frequency than reported here, such as genetic variants in *CHD1L* for 10% of primary CNS lymphomas, *GFM1* for 5.7% of cholangiocarcinomas, *MEIS1* for 5.7% of uterine carcinosarcomas, and *NFX1* for 6.7% of malignant peripheral nerve sheath tumors [20]. The majority of the recurrent deleterious variants were not found in the control cohort indicating tumor specificity. Two of the identified genes, *NCOR1* and *ASPM*, have previously been identified as mutational cancer driver genes (high confidence driver) by Tamborero *et al*. [21].

Of the 52 different genes harboring recurrent deleterious variants found in at least one of the histotype or survival groups, frameshift insertion in the *UBR5* gene was the only deleterious variant that has previously been associated with cancer according to the COSMIC database. This finding suggests that the present study identified novel deleterious variants associated with cancer. Moreover, novel mutation signatures in relation to histotypes were identified. Among these, increased DNA methylation of the *CASP8AP2* gene has previously been associated with less sensitivity to cisplatin and taxol in a breast cancer cell line (MDA-MB-231) [22], elevated *UBR5* gene expression levels have been observed in cisplatin-resistant ovarian cancer patients in comparison with cisplatin-responsive patients [23], and *SEMA4D* has been shown to play a role in progression of multiple cancers, such as ovarian cancer [24]. The *IPMK* gene has been shown to be involved in cell cycle arrest and apoptosis in ovarian cancer [25] and the *BECN1* gene has been suggested to be a tumor-suppressor gene and its expression levels to be associated with ovarian cancer prognosis [26]. To our knowledge, no association with ovarian cancer has previously been found for the genetic variants in the *CLEC2B*, *ACACA*, *OSTM1* and *SLC28A2* genes. As next-generation sequencing analyses become more commonly used in the clinic, these novel mutation signatures may help to improve subclassification of the histotypes. The deleterious variant in the *OSTM1* gene may be of prognostic importance, since it showed significantly higher mutation rates in the 0-2 year survival group compared to the other survival groups.

Several of the genes comprising the deleterious variants also had an effect on gene expression patterns. Significant overexpression of *AP2M1*, *GFM1*, *HIST1H1E*, and *ZNF148,* and underexpression of *BECN1* and *NCOR1* were identified in HGSC compared to the other histotypes. Out of these genes, only the *AP2M1* gene (DNA amplification) has previously been associated with HGSC [27]. Moreover, the *SEMA4D* gene was significantly overexpressed, and the *ARV1* gene was significantly underexpressed in CCC in comparison with the EC and MC histotypes. With regard to survival groups, *ALMS1* and *VWA3A* may be used as prognostic biomarkers, since they were significantly underexpressed in the 0-2 year survival group compared to the 2-5 year and >10 year survival groups, and 2-5 year and >10 year survival groups, respectively, wherein the *VWA3A* gene has previously been associated with survival [28]. However, the observed changes in gene expression pattern were primarily associated with specific histotype or survival groups rather than a result of the genetic variant. These findings indicate that the gene expression patterns were influenced by other molecular mechanisms than the genetic variation in specific indels (insertions/deletions) and SNVs (single-nucleotide variants). Of the 15 genes showing significant changes in gene expression patterns in the presence of potential deleterious variants, *SLC28A2* was the only gene with differential gene expression patterns that could be attributed to mutation status in the MC histotype, as well as, between variant carriers in the HGSC and MC histotypes. Consequently, only histotype-specific changes in gene expression were observed for the remaining genes containing genetic variants, which may suggest that histotype and/or other molecular mechanisms play a role in determining the gene expression levels for these genes. In the survival groups, *ARCN1* showed a significant difference in gene expression patterns between variant carriers in the 0-2 year and 5-10 year survival groups, suggesting that *ARCN1* may have a tumor suppressor effect as elevated expression thereof indicated longer survival times. The deleterious frameshift insertion in *MTUS1* was particularly interesting since it resulted in a significant difference in both gene expression patterns and overall survival compared to non-variant carriers. The elevated gene expression levels in variant carriers compared to non-variant carriers caused by the frameshift insertion may be explained in that the *MTUS1* degradation site may have been removed. Interestingly, a protective effect for overall survival was shown for the deleterious variant in *MTUS1*, suggesting that the genetic variant has a tumor-suppressor effect. Although the tumor suppressor effect of *MTUS1* has been shown in multiple cancer forms [29–31], this is the first report to our knowledge correlating both *MTUS1* gene expression and mutation status with overall survival.

Fusion events may play an important role in the development of epithelial cancers, since they may be strong driver mutations and promote genomic instability [32]. High resolution RNA-seq can be used to identify fusion transcripts that are expressed within tumor tissue. The majority of the identified fusion transcripts had one gene partner spanning non-coding exonic DNA regions, suggesting that the majority of fusion transcripts do not generate a corresponding fusion protein, but may influence the expression of the fusion partner. These fusion transcripts may have arisen by chance due to genomic instability. Only 12.7% of the identified fusion transcripts were predicted to be in coding regions, *e.g.* in-frame, out-of-frame or promoters (5’ UTRs). Out-of-frame fusion transcripts are also assigned to this group since they may be placed back in-frame through alternative splicing or genetic variations. Almost half of the fusion transcripts had an inverted orientation of their fusion partners, indicating fusion transcript formation via inversion events. Interestingly, we identified a higher prevalence of interchromosomal fusion events than intrachromosomal fusion events, which is in contrast to previous reports in cancer [33, 34]. These differences may be the result of multiple interchromosomal fusions on chromosome 11, which predominantly spanned ncRNAs. Moreover, several interesting in-frame kinase fusions were identified which may be targetable with kinase inhibitors.

*MALAT1* lncRNA has been associated with overexpression in various cancers and linked to unfavorable overall survival when overexpressed [35]. *MALAT1* was significantly overexpressed in the study cohort compared to the normal controls, which is consistent with recent studies linking *MALAT1* overexpression with significantly increased cell proliferation and invasion in ovarian cancer [36, 37]. To our knowledge, *MALAT1* has not previously been reported to be involved in fusion events in ovarian carcinomas. *MALAT1* was highly promiscuous (forming fusions with multiple partners) in the present study, suggesting that the majority of the fusions occurred at the RNA level. Nevertheless, FISH analysis demonstrated that several *MALAT1* fusions also occur at the DNA level. However, the fusions were observed in few cells indicating extensive clonal heterogeneity. The most commonly identified fusion transcript across the study cohort, *AHNAK-MALAT1*, was found in 25.8% (n=24) of the samples and this frequency is even higher (35.4%, n=34) if the reciprocal fusion transcript (*AHNAK-MALAT1*, *MALAT1-AHNAK*) is taken into account. These numbers are extremely high considering the heterogeneous nature of ovarian carcinomas. Few fusion transcripts involving *MALAT1* were specific for a particular histotype or survival group. Previous reports have identified several fusion transcripts in HGSC, including *BCAM-AKT2* (7%), *CDKN2D-WDFY2* (20%) and *ESRA-C11orf20* (15%), wherein the majority of the samples were in late-stages, but absent in the present study [38–40]. A further study identified nine recurrent fusion transcripts (present in at least 2/220), whereof two fusion transcripts *CRHR1-KANSL1* and *COL14A1-DEPTOR* were also found in the present study cohort [41].

Oncofuse identified 105 potentially oncogenic fusion transcripts. Six of the most common fusion partners containing the *MUC16*, *AHNAK*, *SYNE2*, *COL3A1*, *COL1A2* and *MACF1* genes were identified as significant driver fusion transcripts. *MALAT1* was not present as a fusion partner among the 105 driver fusion transcripts. This may suggest that *MALAT1* has not previously been identified as a fusion transcript and further research is necessary to elucidate its role in ovarian tumorigenesis. However, Lanzós *et al.* [42] recently identified *MALAT1* as a high confidence candidate of being a cancer driver lncRNA.

In the gene expression analysis, 23 genes were found to be differentially expressed in at least two of the survival group comparisons, and associated with tumor aggressiveness. These genes may be seen as common cancer genes rather than ovarian cancer specific genes associated with tumor aggressiveness, since the histotypes have different origin and clinical behavior. Interestingly, the majority of the differentially expressed genes was involved in cancer-related pathways and/or were associated with cancer, such as female genital tract cancer. For example, *ARG2* and *MMP1* expression have been found to correlate with poor prognosis in gastric and ovarian cancer, respectively [43, 44]. *MTRNR2L1* was significantly underexpressed in both the 2-5 year survival group and 5-10 year survival group compared with long-term survivors (only HGSC samples taken into account). Little is known about the function of the *MTRNR2L1* gene and it has not previously been associated with HGSC.

In summary, we have identified several novel genetic aberrations associated with histotype and/or clinical outcome for early-stage ovarian carcinomas. In addition, several potential diagnostic and prognostic biomarkers were identified. Potential diagnostic biomarkers include the recurrent deleterious tumor-specific genetic variants which were only found in the study cohort but absent in the normal controls, among which 15 genetic variants were identified with significant differences in mutation rates between the histotype groups, *e.g.* frameshift insertion in *OSTM1* (higher mutation rates in MC compared with the other histotype groups), frameshift deletion in *BECN1* (lower mutation rates in CCC compared with the other histotype groups), and absence of the frameshift insertion in *SEMA4D* in MC. In addition, the *SLC28A2* gene showed a significant difference in gene expression patterns between variant and non-variant carriers in the MC histotype. Lastly, *MALAT1* expression may also be used as a diagnostic biomarker as it was significantly higher in the study cohort compared to the normal controls. Potential prognostic biomarkers include the recurrent deleterious tumor-specific genetic variants which differed between the survival groups, *e.g.* frameshift insertion in *OSTM1* (higher mutation rates in 0-2y compared with the other survival groups). The frameshift insertion in *MTUS1* presented a potential tumor-suppressor effect due to the mutation-dependent gene expression patterns and its protective effect on OS in variant carriers. Moreover, the 23 differentially expressed genes associated with overall survival (tumor aggressiveness) may be used as prognostic biomarkers. Further studies need to be performed in order to validate the clinical significance these aberrations may have on ovarian carcinoma subclassification and patient stratification using *e.g.* immunohistochemistry and *in vivo* functional studies.

## MATERIALS AND METHODS

### Patients and tumor samples

Primary invasive ovarian carcinomas from 96 patients diagnosed between 1994 and 2006 were obtained from the fresh-frozen tumor bank at the Sahlgrenska University Hospital Oncology lab (Gothenburg, Sweden). Clinicopathological characteristics and overall survival data were obtained from the National Quality Registry at the Regional Cancer Center West (Gothenburg, Sweden) and the Cancer Registry at the National Board of Health and Welfare, respectively. Of the available fresh-frozen samples in the tumor bank, patients were chosen for inclusion in the study cohort according to the International Federation of Gynecology and Obstetrics (FIGO) stage I and II, as well as overall survival calculated from the date of initial diagnosis to the date of death of any cause and stratified into four survival groups, i.e. 0-2 years, 2-5 years, 5-10 years and >10 years.

The tumor specimens were reclassified according to current WHO criteria [1–3, 45] with regard to histotype and histological grade by pathologists at Sahlgrenska University Hospital using four micrometer full-face FFPE sections stained with hematoxylin and eosin. The histotype distribution in the present study was relatively consistent with previous reports. However, a slightly higher number of HGSC samples (52%) and lower number of EC (18%) and CCC (18%) were used in the present study than has previously been reported for early-stage ovarian carcinomas (HGSC (35.5%), LGSC (1.9%), EC (26.6%), MC (7.5%), CCC (26.2%)) [46]. All procedures were performed in accordance with the Declaration of Helsinki and approved by the Regional Ethical Review Board (Gothenburg, Sweden; case number 767-14). The Regional Ethical Review Board approved a waiver of written consent to use the tumor specimens. The clinicopathological features of the 96 cases with regard to the histotype reclassification are shown in Table 1 and overall survival in Supplementary Table 4. All 96 patients underwent laparotomy and debulking cytoreductive surgery.

**Table 1:** Clinicopathological characteristics of the 96 patients (grouped by histotype) with ovarian carcinoma

	No. of patients (%)						
	All	Histotype					P-value
		*HGSC*	*LGSC*	*EC*	*MC*	*CCC*	
All	96	50 (52)	1 (1)	17 (18)	11 (11)	17 (18)	
**Mean age**							NA
*mean age (range)*	63 (25-86)	63 (32-86)	78	64 (25-83)	61 (39-80)	63 (42-84)	
**Overall survival**							0.118
*0-2y*	9 (9)	2 (4)	1 (100)	1 (6)	3 (27)	2 (12)	
*2-5y*	27 (28)	17 (34)	NA	5 (29)	2 (18)	3 (18)	
*5-10y*	33 (34)	18 (36)	NA	5 (29)	3 (27)	7 (41)	
*>10y*	27 (28)	13 (26)	NA	6 (35)	3 (27)	5 (29)	
**Cause of death**							0.035
*Ovarian carcinoma*	48 (50)	32 (64)	1 (100)	3 (18)	2 (18)	10 (59)	
*Other cancer*	13 (14)	7 (14)	0	3 (18)	3 (27)	0	
*Other*	21 (22)	5 (10)	0	6 (35)	4 (36)	6 (35)	
*Not available*	1 (1)	0	0	0	0	1 (6)	
Alive^*^	13 (14)	6 (12)	0	5 (29)	2 (18)	0	
**Stage**							0.145
*I*	64 (67)	29 (58)	NA	12 (71)	9 (82)	14 (82)	
*II*	32 (33)	21 (42)	1 (100)	5 (29)	2 (18)	3 (18)	
**Tumor grade EC**							NA
*FIGO grade I*	2 (2)	NA	NA	2 (12)	NA	NA	
*FIGO grade II*	9 (9)	NA	NA	9 (53)	NA	NA	
*FIGO grade III*	6 (6)	NA	NA	6 (35)	NA	NA	
Dualistic model^**^							<0.001
*Type I*	46 (48)	0	1 (100)	17 (100)	11 (100)	17 (100)	
*Type II*	50 (52)	50 (100)	0	0	0	0	
**CA125**							0.094
*<35*	26 (27)	8 (16)	0	7 (41)	5 (45)	6 (35)	
*35-65*	16 (17)	13 (26)	0	0	2 (18)	1 (6)	
*>65*	54 (56)	29 (58)	1 (100)	10 (59)	4 (36)	10 (59)	
**Ploidy**							0.213
*near diploid*	25 (26)	15 (30)	0	7 (41)	2 (18)	1 (6)	
*aneuploid*	69 (72)	35 (70)	1 (100)	9 (53)	8 (73)	16 (97)	
*Not available*	2 (2)	0	0	1 (6)	1 (9)	0	
**Chemotherapy**							0.315
*Yes*	95 (99)	49 (98)	1 (100)	17 (100)	11 (1)	17 (100)	
*No*	0	0	0	0	0	0	
*Not available*	1 (1)	1 (2)	0	0	0	0	

^*^ Alive per 2016.01.01.

^**^ Dualistic model according to Kurman, R.J. *et al*., The Dualistic Model of Ovarian Carcinogenesis, Revisited, Revised, and Expanded, 2016.

### Control cohort

Approval for access to the Cancer Genome Atlas (TCGA) genomic data was obtained through the database of Genotypes and Phenotypes (dbGaP; project #11044). Raw sequencing data for 30 normal ovarian solid tissue samples (control cohort; Supplementary Table 5) was retrieved from TCGA-OV data collection through the Genomic Data Commons Data Portal (GDC Data Portal) [18, 47]. The normal controls had been analyzed by whole exome sequencing (WXS) and mapped against GRCh38. To minimize batch differences between the study cohort and the control cohort, the raw data for the control cohort was converted to FASTQ format using the BEDTools (v. 2.25.0) module and compressed using the gzip module in Uppsala Multidisciplinary Center for Advanced Computational Science (UPPMAX). The FASTQ files were thereafter processed using the same pipeline as the study cohort for variant calling and differential gene expression analysis (see below).

### Whole-transcriptome RNA-seq

Total RNA was extracted from the tumor samples using the RNeasy Lipid Tissue Mini Kit (Qiagen), followed by RNA concentration and integrity assessment using Nanodrop ND-1000 (Nanodrop Technologies) in combination with QuBit (ThermoFisher Scientific) and the RNA 6000 Nano LabChip Kit with Agilent 2100 Bioanalyzer (Agilent Technologies), respectively. Samples with an RNA integrity number (RIN) of 6 or higher were processed at the Science for Life Laboratory (National Genomics Infrastructure, Stockholm). Illumina TruSeq strand-specific RNA libraries (Ribosomal depletion using RiboZero human) containing 125 bp pair-end reads were obtained for each sample on a HiSeq2000 sequencer (Illumina). Read alignment yielded approximately 9.7 to 22.3 million aligned reads per sample (median, 19.4 million aligned reads). Computations were performed on resources provided by SNIC through UPPMAX under Project SNIC b2015239.

#### Quality control of raw RNA-seq data

A quality control check of the FASTQ files was performed using default FastQC parameters (v. 0.11.2) and compiled using MultiQC (v. 0.6). Low quality bases (Phred quality scores below 20) and adapter sequences were removed utilizing the TrimGalore wrapper script (v. 0.4.0). FastQC and MultiQC were thereafter performed on the trimmed FASTQ files examining the quality of the trimmed reads and ensuring that all adapter sequences had been removed [48]. A principal component analysis (PCA) was performed on the variance stabilizing transformation data (vsd) values on raw gene counts for each sample using DESeq2 (v. 1.14.0) in R/Bioconductor (v. 3.2.5).

#### Variant calling

Genetic variants were identified according to the Genome Analysis Toolkit (GATK) (v. 3.6) Best Practices protocol, Broad Institute (https://software.broadinstitute.org/gatk/guide/article?id=3891). More specifically, the trimmed FASTQ files were aligned to the human reference genome hg19 (GRCh37) using the STAR (v. 2.5.0c) aligner in a two-pass approach [49]. MultiQC was run on Log.final.out-files received from the mapping with STAR to control the mapping quality of each sample. One sample had below 80 % uniquely mapped reads, and was hence removed from the study cohort (OV386 = 52.4% uniquely mapped reads). This sample is not accounted for under the “Patients and tumor samples” section above.

In brief, the GATK SplitNCigarReads tool was used to remove false positive calls caused by splicing inaccuracies from the STAR aligner. Base quality score recalibration (BQSR) was performed using GTF files (dbsnp_138.hg19.vcf, dbsnp_138.hg19.vcf.idx, Broad Institute). The genetic variants were called using the HaplotypeCaller. Hard filters were applied filtering clusters of at least three SNPs, which were in a window of at least 35 bases and based on Fisher Strand (FS>30) and quality by depth (QD<2). The filtered genetic variants were annotated with ANNOVAR [50] and thereafter further filtered with the 1000 Genomes Project dataset (1000g2015aug) [51, 52] and dbSNP (hg19_snp138) with a minor allele frequency (MAF) threshold of 0.01 to remove common genetic variants found in the human population. Potential deleterious variants predicted to have an effect on the amino acid sequence were identified by filtering conservatively for the Sequence Ontology terms frameshift insertion (SO:0001909), frameshift deletion (SO:0001910), stopgain (SO:0001587), or stoploss (SO:0001578) [53, 54]. The genetic variants were matched against the Catalogue of Somatic Mutations in Cancer (COSMIC) database (v.70, Aug. 2014) [55] to annotate known genetic variants associated with cancer.

#### Differential gene expression analysis

Raw read counts, *i.e.* the number of sequences that map to each transcript of the human reference genome (hg19 assembly), were calculated using htseq-count in the htseq (v. 0.6.1) module in UPPMAX on name sorted STAR (1-pass mode) BAM files. Fragments per kilobase of exon per million fragments mapped (FPKM) values were computed by running the Cufflinks (v. 2.2.1) module on STAR (1-pass mode) BAM files sorted by coordinates. Differentially expressed transcripts were determined using normalized count values of the tumor samples in the study and control cohort. DESeq2 (v. 1.14.0) in R/Bioconductor (v. 3.2.5) was used to compare gene expression levels between patients belonging to different histotype and/or survival groups to identify the differentially expressed transcripts.

#### Identification and validation of fusion transcripts

FusionCatcher [56] (v. 0.99.5a) with the associated databases ENSEMBL, UCSC and RefSeq was used to identify fusion transcripts. FusionCatcher implemented Bowtie (v. 0.12.6), BLAT (v. 35), STAR and Bowtie2 (v. 2.2.3) to identify fusion junctions and align the fusion transcripts to the GRCh37 human reference genome assembly. Identified fusion transcripts marked with fusion descriptions indicating fusion genes of high or very high probability of being a false positive fusion transcript were removed [57].

The Oncofuse tool [58] was used to predict the oncogenic potential of the identified fusion transcripts across the study cohort (tissue type: EPI (epithelial origin)). A functional prediction score was assigned to each fusion transcript describing the probability of the fusion transcript being a driver event (Bayesian probability scores < 0.5).

### Fluorescence *in situ* hybridization (FISH)

Dual-color interphase FISH was performed using a selection of recurrent fusion transcripts across the study group identified by FusionCatcher. More specifically, bacterial artificial chromosome (BAC) clones covering each fusion partner were selected from BACPAC Resources Center, Children's Hospital Oakland Research Institute, CA, USA (Supplementary Table 6) and validated on normal metaphase chromosome preparations. BAC DNAs corresponding to each fusion transcript were extracted (Qiagen Plasmid Maxi kit) and separately labeled for dual-color FISH by nick translation with biotin-16-dUTP (detected by FITC) and digoxigenin-11-dUTP (detected by Rhodamine) (Roche Diagnostics, Mannheim, Germany), respectively. Touchprint preparations were prepared from fresh-frozen tumors corresponding to the RNA sequenced ovarian tumor samples. Biotin-16-dUTP and dioxigenin-11-dUTP labeled probes were co-hybridized to denaturated interphase nuclei slides overnight at 37 °C. The hybridized slides were counterstained with DAPI and mounted in an antifade solution (Vectashield DAPI, Vector Laboratories, Burlingame, CA, USA). Sample evaluation and image acquisition were performed using a Leica DMRA2 fluorescent microscope (Leica, Leica Microsystems, Wetzlar, Germany) equipped with an ORCA Hamamatsu CCD (charged-couple devices) camera and filter cubes specific for green fluorescein isothiocyanate (FITC), red rhodamine, and UV for blue 4′,6′-diamidino-2′-phenylindole dihydrochloride (DAPI) counterstain visualization. Image preparation was performed using CW4000 software, QFISH.

### Ingenuity pathway analysis (IPA)

Pathway analysis was performed using Ingenuity Pathway Analysis (Ingenuity Systems, Redwood City, USA) to identify cancer-related biological functions associated with the identified genetic variants, differentially expressed genes and fusion transcripts. The biological functions were generated using Fisher's exact test (*P*<0.05).

### Genome-wide SNP genotyping

Genomic DNA was extracted (Wizard Genomic DNA Purification Kit, Promega) followed by phenol chloroform purification with Phase Lock Gel Light (5 Prime) for nine samples in the study cohort comprising a FISH verified fusion transcript. Genome-wide SNP genotyping analysis (Illumina Infinium HumanOmni2.5-8 v. 1.3 Beadchips) was performed on the purified DNA at the SCIBLU Genomics DNA Microarray Resource Center (SCIBLU), Department of Oncology, Lund University.

The identified genetic variants and fusion transcripts were correlated to copy number alterations and DNA breakpoints present in the SNP genotyping data. The RNA-seq (genetic variants and fusion transcripts) and SNP genotyping data were visualized as circos plots using the Circos (v. 0.66) module in UPPMAX.

### Statistical analyses

A comparison of genetic variant frequency and gene expression levels dependent on genetic variants or fusion transcripts was performed between the study and control cohorts, histotype and/or survival groups using a 0.05 p-value cutoff in Microsoft Excel with the TTEST function or analysis of variance (ANOVA) in R/Bioconductor (v. 3.2.5) as appropriate. All p-values are two-sided. Survival rates according to mutation status were calculated with Kaplan-Meier curves and tested with log-rank test (survival, v. 2.40-1). Univariate Cox proportional hazard models were calculated for mutation status using DSS (the time from initial diagnosis to ovarian cancer-related death) and OS (the time from initial diagnosis to death from any cause). Statistical significance is indicated as ^*^P<0.05, ^**^P<0.01 or ^***^P<0.001.

### Data availability

The RNA-seq and SNP genotyping data have been deposited in the NCBI Gene Expression Omnibus (http://www.ncbi.nlm.nih.gov/geo/) under accession number GSE101109.

## SUPPLEMENTARY MATERIALS FIGURES AND TABLES












