# Supplementary material for: Transcriptomic and genomic profiling of early-stage ovarian carcinomas associated with histotype and overall survival
Source: Oncotarget. 2018 Oct 12;9(80):35162–80. doi: 10.18632/oncotarget.26225 (PMC6205557; doi:10.18632/oncotarget.26225)
Supplement: Supplementary file 1 [file oncotarget-09-35162-s001.pdf]

Transcriptomic and genomic profiling of early-stage ovarian carcinomas associated with histotype and overall survival

SUPPLEMENTARY MATERIALS

Supplementary Table 1A: Significance (two-tailed *t*-test) of the deleterious variants presented in Figure 2A. Significant comparisons (*P*<0.05) are marked in bold.

See Supplementary File 1

Supplementary Table 1B: Significance (two-tailed *t*-test) of the deleterious variants presented in Figure 2B. Significant comparisons (*P*<0.05) are marked in bold.

See Supplementary File 2

Supplementary Table 2A: Significance (two-tailed *t*-test) of gene expression profiles associated with the genes presented in Figure 2A. Significant (*P*<0.05) comparisons are marked in bold.

See Supplementary File 3

Supplementary Table 2B: Significance (two-tailed *t*-test) of gene expression profiles associated with the genes presented in Figure 2B. Significant (*P*<0.05) comparisons are marked in bold.

See Supplementary File 4

Supplementary Table 3: Predicted biological functions of driver fusion transcripts identified by Oncofuse.

See Supplementary File 5

Supplementary Table 4: Clinicopathological characteristics of the 96 patients (grouped by total survival) with ovarian carcinoma

	All	No. of patients (%)				P-value
		Overall survival				
		0-2y	*2-5y*	*5-10y*	*>10y*	
All	96	9 (9)	27 (28)	33 (34)	27 (28)	
**Mean age**						0.342
mean age (range)	63 (25-86)	66 (49-80)	65 (32-84)	64 (42-86)	59 (25-79)	
**Histotype**						0.118
HGSC	50 (52)	2 (22)	17 (63)	18 (55)	13 (48)	
LGSC	1 (1)	1 (100)	NA	NA	NA	
EC	17 (18)	1 (11)	5 (19)	5 (15)	6 (22)	
FIGO grade I	2 (2)	0	1 (4)	1 (3)	0	
FIGO grade II	9 (9)	1 (11)	2 (7)	2 (6)	4 (15)	
FIGO grade III	6 (6)	0	2 (7)	2 (6)	2 (7)	
MC	11 (11)	3 (33)	2 (7)	3 (9)	3 (11)	
CCC	17 (18)	2 (22)	3 (11)	7 (21)	5 (19)	
**Cause of death**						0.052
Ovarian carcinoma	48 (50)	6 (67)	18 (67)	19 (58)	5 (19)	
Other cancer	13 (14)	1 (11)	7 (26)	4 (12)	1 (4)	
Other	21 (22)	2 (22)	2 (7)	10 (30)	7 (26)	
Not available	1 (1)	0	0	0	1 (4)	
Alive[*]	13 (14)	0	0	0	13 (48)	
**Stage**						0.598
I	64 (67)	5 (56)	16 (59)	24 (73)	19 (70)	
II	32 (33)	4 (44)	11 (41)	9 (27)	8 (30)	
**Dualistic model**[**]						0.192
Type I	46 (48)	7 (78)	10 (37)	15 (45)	14 (52)	
Type II	50 (52)	2 (22)	17 (63)	18 (55)	13 (48)	
**CA125**						0.125
<35	26 (27)	1 (11)	11 (41)	9 (27)	5 (19)	
35-65	16 (17)	1 (11)	7 (26)	3 (9)	5 (19)	
>65	54 (56)	7 (78)	9 (33)	21 (64)	17 (63)	
**Ploidy**						0.044
near diploid	25 (26)	1 (11)	6 (22)	5 (15)	13 (48)	
aneuploid	69 (72)	8 (89)	20 (74)	28 (85)	13 (48)	
Not available	2 (2)	0	1 (4)	0	1 (4)	
**Chemotherapy**						0.185
Yes	95 (99)	9 (100)	27 (100)	32 (97)	27 (100)	
No	0	0	0	0	0	
Not available	1 (1)	0	0	1 (3)	0	

[*] Alive per 2016.01.01.

[**] Dualistic model according to Kurman, R.J. *et al*., The Dualistic Model of Ovarian Carcinogenesis, Revisited, Revised, and Expanded, 2016.

Supplementary Table 5: Universally unique identifiers (UUIDs) of samples in the control cohort

UUID
40d4dc94-80da-46f7-b760-368efbe4c219
17903d09-c494-400b-8b5c-3c98c72d5401
90e9f7de-8092-4d91-8e94-fccc26dca13b
f0cc3d73-c740-4739-9bd3-45d220d6e9a4
77e268ce-b8cb-456c-9618-8d86594c449c
cc6dcb92-2301-49d3-bcb1-3bc77db2680b
8f26ba07-f20d-4903-870d-336f3522979c
ea3b0737-1bdb-4995-89ec-3e18ab03a08f
e9d1a73a-7347-4357-805f-3e5c3d989afb
f6387370-828d-4410-af3a-772d80f69b26
49052acd-4510-470c-91d6-96a757f77378
ee24349a-6f97-416e-9521-349d4251bdcd
7732de34-8f4a-438a-a9cf-d0ee1f4a574b
4dd02fe1-ea4d-49e6-95d1-f6ead3580206
d205ca76-756f-432d-a0bc-6a2f6a1ad7ea
4206cd29-bc3d-4986-b0c2-36a940843580
1abafcf4-da1d-41db-aa0c-262217093c07
13b358b2-7d22-4c4b-9b76-2bc9c9b9eb1d
1686d945-3487-40be-8c5a-14ea6aa4c709
00ae0345-6ebb-4d98-9344-9641e2e94604
fb56b891-f810-4581-aec9-5db4d67d7e06
19752600-060d-4f66-8db6-f25bc31ae6b0
6726e87d-ff98-45c6-817c-031473f5e20c
360809ca-1bfd-4789-a94a-b129d0e36875
3671c416-4ce0-445b-8d7e-bd8eefd5dc9c
09fea735-4a14-43b2-b0f4-9cec5af5a47c
7c46fa8a-b6de-45b6-8699-89ae5e3434c5
3b46446c-de3d-411d-9fe2-b56c12f72979
494dcd7e-6775-4bb8-874e-5c5f2da5874f
d8a97612-cdb6-40d7-a54c-9e6bd76bffc1

Supplementary Table 6: FISH probe identifiers

Gene	BAC identification number
AHNAK	CTD-2240J20
C3	RP11-330I7
CANX	RP11-305G6
COL1A2	RP11-57C4
COL3A1	RP11-655A7
EIF4A2	RP11-119E13
MACF1	RP11-368G12, RP11-659O9, RP11-434G16
MALAT1	RP11-642F7, RP11-1104L6, RP11-472D15
MUC16	RP11-79F15, RP11-282A10
RMRP	RP11-331F9
RPPH1	RP11-113M16
SYNE2	RP11-729I13, RP11-945L20, RP11-726F7, RP11-14C21
XIST	RP13-183A17
